# Supplementary material for: Enhancing Solid-Phase Extraction of Tamoxifen and Its Metabolites from Human Plasma Using MOF-Integrated Polyacrylonitrile Composites: A Study on CuBTC and ZIF-8 Efficacy
Source: Nanomaterials (Basel). 2023 Dec 26;14(1):73. doi: 10.3390/nano14010073 (PMC10780427; doi:10.3390/nano14010073)
Supplement: Supplementary file 1 [file nanomaterials-14-00073-s001.zip › nanomaterials-2746695-supplementary.pdf]

nanomaterials

Supplementary materials

Enhancing solid-phase extraction of tamoxifen and its metabolites from human plasma using MOF-integrated polyacrylonitrile nano-composites: a study on CuBTC and ZIF-8 efficacy

Domingo R. Flores-Hernandez [1], H. M. Leija Gutiérrez [2], JA Hernandez-Hernandez [3], Jose Antonio Sanchez-Fernandez [4,*] and Jaime Bonilla-Rios [1,*]

[1] Escuela de Ingeniería y Ciencias, Tecnologico de Monterrey, Ave. Eugenio Garza Sada 2501, Monterrey 64849, NL, Mexico; A00819488@tec.mx (D.R.F.-H.); jbonilla@tec.mx (J.B.-R.);
[2] Universidad Autónoma de Nuevo Leon, CICFM-FCFM. Av. Universidad S/N, Ciudad Universitaria, 66451 San Nicolas de los Garza, NL, Mexico; hector.leijagt@uanl.edu.mx (H.M.L.-G.)
[3] Escuela de Medicina y Ciencias de la Salud, Tecnologico de Monterrey, Monterrey 64710, NL, Mexico; j.a.hernandez@tec.mx (J.A.H.-H.)
[4] Procesos de Polimerización, Centro de Investigación en Química Aplicada, Blvd. Enrique Reyna No. 140, Saltillo, 25294, CH, Mexico; antonio.sanchez@ciqa.edu.mx (J.A.S.-F.)

* Correspondence: jbonilla@tec.mx; Tel.: +52-81-8358-2000 (ext. 5721); antonio.sanchez@ciqa.edu.mx

Solutions preparation for UPLC-Ms/Ms analysis

In preparation for the study, stock solutions of the analytes and internal standards were prepared by dissolving them in methanol at a concentration of 1 mg/ml for the isomers as reported by Bubin-Dubigeon et. al [1]. These stock solutions were then diluted in a mixture of water and methanol (7/3) containing 0.1% formic acid to ensure proper solubilization of the analytes. The dilution ranges for each compound were as follows: TAM and NDTAM, 5000, 2000, 1000, 500, 200, 100, 50, 20, and 10 ng/ml; ENDO, 1000, 400, 200, 100, 40, 20, 10, and 4 ng/ml; and 4OHTAM, 500, 200, 100, 50, 20, 10, 5, 2, and 1 ng/ml.

The diluted solutions mentioned above were further diluted in blank plasma at the time of analysis to generate calibrator concentrations. The calibrator concentrations for TAM and NDTAM were 500, 200, 100, 50, 20, 10, 5, 2, and 1 ng/ml. For ENDO, the concentrations were 100, 40, 20, 10, 4, 2, 1, and 0.4 ng/ml. Lastly, for 4OHTAM, the concentrations were 50, 20, 10, 5, 2, 1, 0.5, 0.2, and 0.1 ng/ml.

Similarly, internal standard solutions were also diluted extemporaneously in acetonitrile with 0.1% formic acid. This yielded final concentrations of 5 and 20 ng/ml for ENDO and OHTAM, and for TAM and NDTAM, respectively.

For the preparation of quality control (QC) samples, separate stock solutions were prepared using the same method as described above. These QC samples were then diluted in plasma to achieve the following concentrations: TAM and NDTAM - 1, 2.5, 40, and 400 ng/ml; ENDO - 0.2, 0.5, 8, and 80 ng/ml; and 4OHTAM - 0.1, 0.25, 4, and 40 ng/ml.

All the stock solutions, intermediary solutions, and QC samples were divided into smaller aliquots and stored at a temperature of -20°C for future use and preservation, a representation the samples preparation is presented in Figure S1.

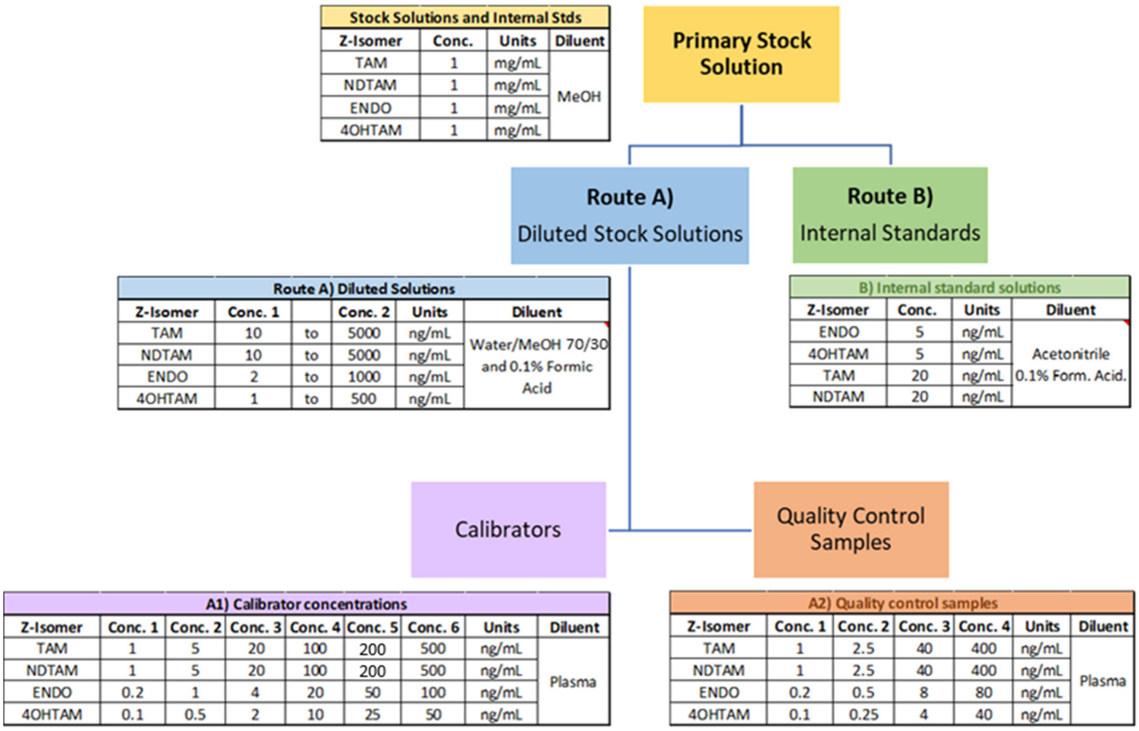

Figure S1. Procedure for preparing calibrators, internal standards and quality control samples for UPLC-Ms/Ms characterization.

Table s1 presents relevant predicted properties of the four analytes: TAM, NDTAM, ENDO, and 4OHT. This table helps understand the potential behavior of these compounds under various solid-phase extraction (SPE) conditions.

Table S1 Predicted physicochemical properties of TAM, NDTAM, ENDO, and 4OHT [26].

Predicted Properties	TAM	NDTAM	ENDO	4OHT
Water Solubility, mg/mL	0.00102	0.000371	0.00092	0.00303
logP	5.93	5.5	5.32	5.44
pKa (Strongest Acidic)	8.76	9.51	8.77	9.11
Hydrogen Acceptor Count	2	2	3	3
Hydrogen Donor Count	0	1	2	1
Polar Surface Area, Å^2	12.47	21.26	41.49	32.7
Polarizability, Å^3	44.19	42.51	43.54	45.44

Solvent based protein precipitation sample pretreatment

Figure S2 depicts the detailed procedure for preparing human blood plasma samples for the analysis of Tamoxifen (TAM), N-desmethyltamoxifen (NDTAM), Endoxifen (ENDO), and 4-Hydroxytamoxifen (4OHT) using protein precipitation methods with methanol and acetonitrile.

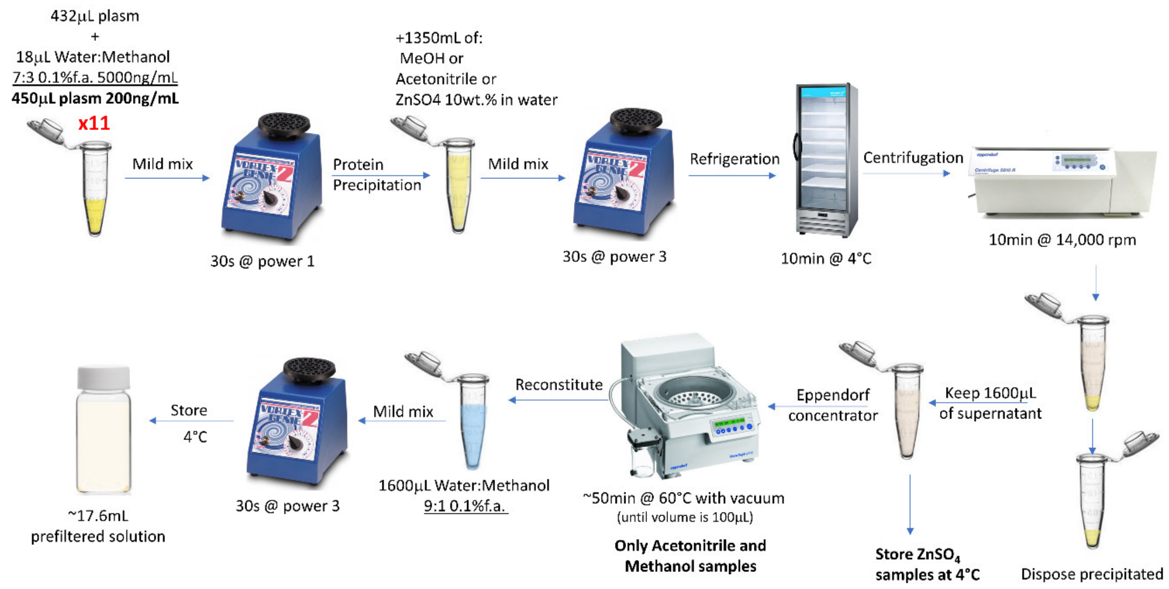

Figure S2 Sample Processing Workflow for ZnSO4, Acetonitrile, and Methanol Precipitated Plasma Samples.

The process initiates with 432 µL of human blood plasma, to which 18 µL of a 70:30 water:methanol solution containing 5000, 5000, 1000, and 500 ng/mL of TAM, NDTAM, ENO, and 4OHT respectively is added. This results in final plasma concentrations of 200, 200, 40, and 20 ng/mL for each compound. The samples are then mildly shaken for 30 seconds in a vortexer to ensure uniform mixing. Following the protocol recommended in [2], a 1:3 sample-to-precipitator ratio is employed, adding 1350 µL of either methanol or acetonitrile to each sample for optimal protein precipitation. After a further 30 seconds of vortexing, the samples are refrigerated at 4°C for 10 minutes to facilitate complete protein denaturation. Subsequently, the samples undergo centrifugation at 14,000 rpm for 10 minutes, separating the precipitated proteins from the supernatant. The supernatant, about 1600 µL, is then collected and concentrated by heating at 60°C for 50 minutes until the volume reduces to approximately 100 µL. Finally, the concentrated samples are reconstituted in a 9:1 water:methanol solution with 0.1% v/v formic acid, mildly shaken for 30 seconds, and stored at 4°C for further analysis. It is crucial to note that all tests are performed within 48 hours of sample preparation to maintain sample integrity.

For samples precipitated using 10 wt.% ZnSO4 in water, reconstitution is not performed, as the salts will not evaporate, and reconstitution would result in the same salt concentration. Therefore, after being vortexed, the supernatant is collected and stored at 4°C.

Chromatographic and mass spectrometer conditions

The MS/MS analysis was conducted using the positive ion ESI mode. Parameters were set as follows: a dessolvation temperature of 650°C, cone gas flow at 20 l/h, and an ion spray voltage of 2 kV. The cone voltage was maintained at +30 V for the molecules under study. Dwell times were set at 0.005 seconds. Nitrogen served as both the nebulizing and curtain gas with a flow rate of 800 l/h. Collision was facilitated using argon. The autosampler injector temperature was 10°C, and the source temperature was set at 150°C. The quantification was done using the multiple reactions monitoring mode, and the specific MS/MS settings can be found in 2. Calibration curves were established by comparing the peak area ratios of the analytes to their internal standards against their known concentrations, using a weight factor of 1/concentration.

Table S2. Retention times and multiple reactions monitoring transitions for TAM, NDTAM, ENDO, and 4OHT metabolites and internal standards TAM-d5, ENDO-d5, and 4OHT-d5

Analyte	Retention time (min)	Parent (m/z)	Quantification transition (m/z)	Confirmation transition (m/z)	Cone voltage (V)	Collision energy (eV)
Tamoxifen	1.81	372.25	72.20	129.25	15	25
Tamoxifen-d5	1.81	377.25	72.20	129.10	15	25
Ndesmethyltamoxifen	1.76	358.00	57.98	90.95	30	23
Z/E-Endoxifen	1.29	374.25	58.10	129.20	15	22
Endoxifen-d5	1.29	379.25		152.00	45	21
4-Hydroxytamoxifen	1.37	388.30	72.10	129.10	20	25
4-Hydroxytamoxifen-d5	1.37	393.30	72.10	129.25	20	25

An analytical column of Acquity UPLC BEH C18 (50 × 2.1 mm, 1.7 μm) was employed. Mobile phase A consisted of water with 0.1 v.% formic acid, while mobile phase B contained acetonitrile with 0.1 v.% formic acid. The starting condition was set at 80% A and 20% B. This was followed by a linear gradient that increased from 20% to 95% of solvent B over 2.5 minutes, with a flow rate of 0.6 ml/min. After this, the initial conditions were reestablished within 0.5 minutes. To cleanse the column, these conditions were sustained for an additional 0.5 minutes. The autosampler and column temperatures were set at 10°C and 40°C, respectively. The injection volume was 6μl, and the entire analysis had a runtime of 3.5 minutes. The chromatographic software's representation of this method can be seen in Figure S3.

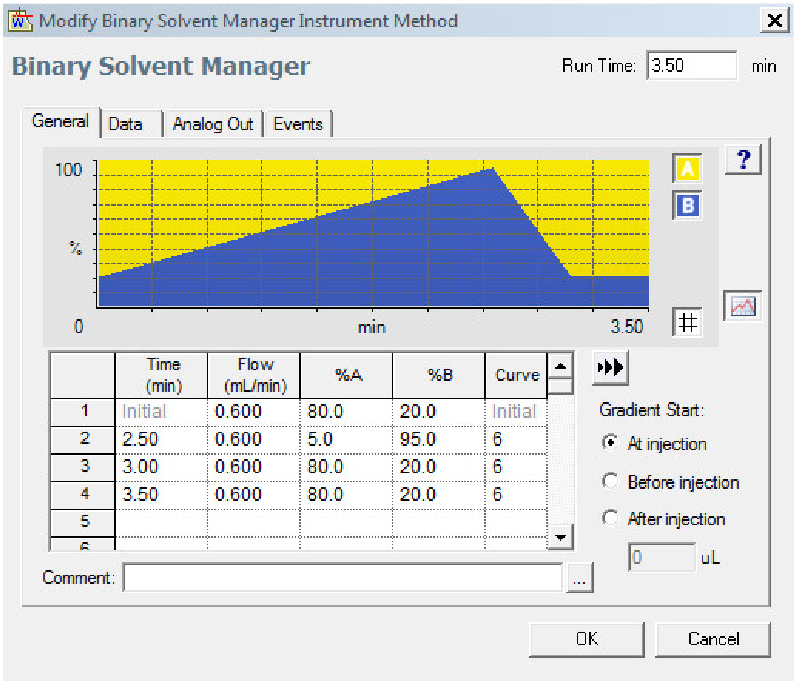

Figure S3 Binary solvent gradient conditions for chromatography

Under the specified chromatographic conditions using the Acquity UPLC BEH C18 column and the detailed gradient program, the chromatograms obtained showcased well-resolved peaks with minimal baseline noise. The mobile phases, consisting of water and acetonitrile both with 0.1 v.% formic acid, provided optimal separation of the analytes. The linear gradient transition from 20% to 95% of solvent B over 2.5 minutes ensured that each compound had a distinct retention time, facilitating easier identification and quantification. When viewed under the mass spectrometer conditions, the peaks exhibited sharpness and clarity, indicating a high level of sensitivity and precision in the detection. The combination of the UPLC conditions with the mass spectrometer settings resulted in chromatograms that were both reliable and reproducible, essential for accurate analysis. Figure S4 displays chromatograms derived from UPLC-MS/MS analysis of Tamoxifen (TAM), N-desmethyltamoxifen (NDTAM), Endoxifen (ENDO), and 4-hydroxytamoxifen (4OHT). Each compound exhibits distinct signal peaks with retention times around 1.78 minutes for TAM, 1.73 minutes for NDTAM, 1.3 minutes for ENDO, and 1.35 minutes for 4OHT. Notably, the chromatogram for ENDO reveals two peaks at 1.28 and 1.32 minutes, indicating that the ENDO metabolite is a 50:50 blend of E/Z isomers. The samples used in this analysis were prepared using methanol dilutions to ensure the equipment was optimally tuned and calibrated.

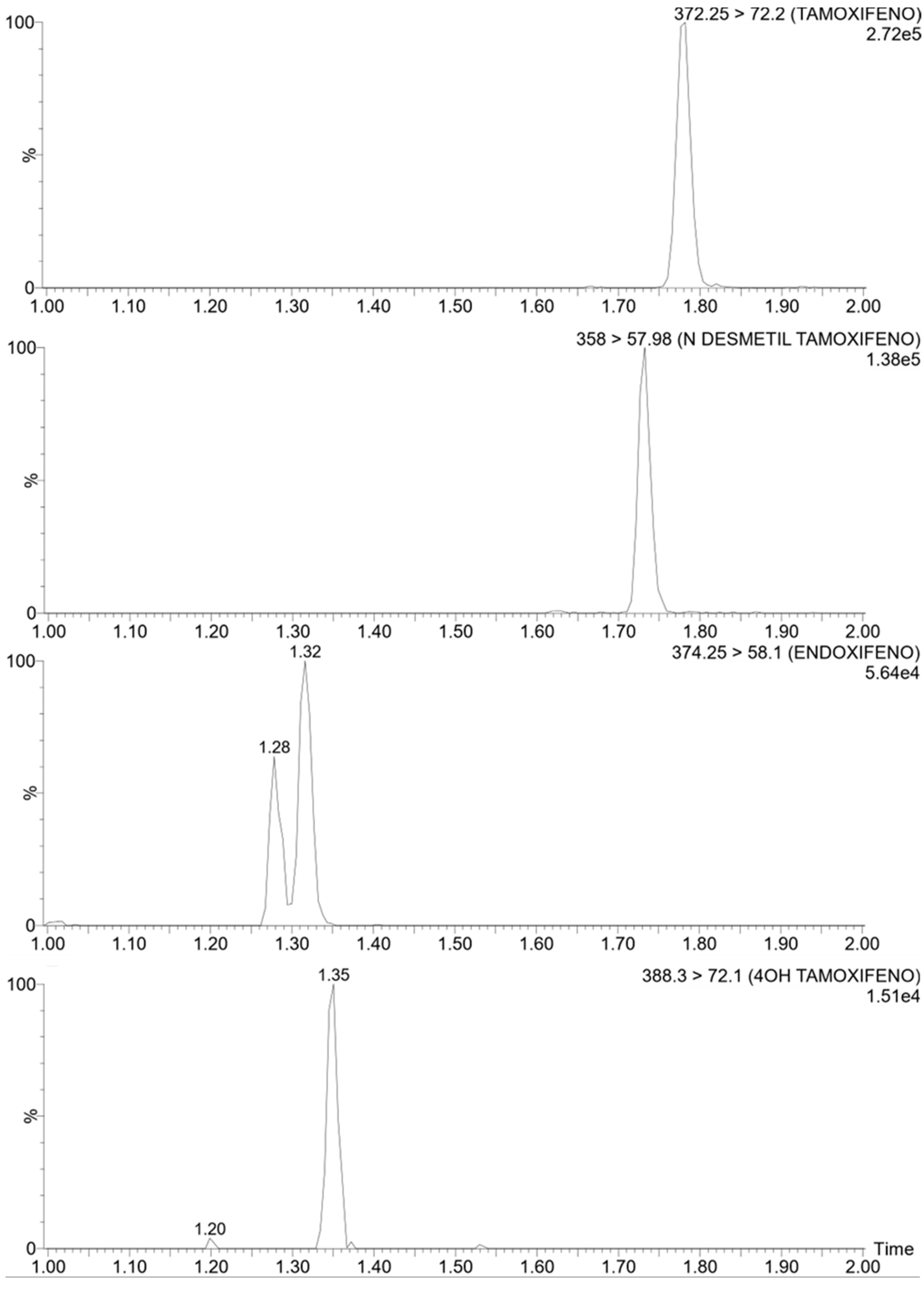

Figure S4. Chromatograms from UPLC-MS/MS Analysis of Tamoxifen and its metabolites

References

1. Bobin-Dubigeon, C.; Campone, M.; Rossignol, E.; Salaun, E.; Amiand, M.B.; Bard, J.M. New UPLC-MS/MS Assay for the Determination of Tamoxifen and Its Metabolites in Human Plasma, Application to Patients. *Future Sci OA* **2019**, *5*.
2. Polson, C.; Sarkar, P.; Incledon, B.; Raguvaran, V.; Grant, R. Optimization of Protein Precipitation Based upon Effectiveness of Protein Removal and Ionization Effect in Liquid Chromatography-Tandem Mass Spectrometry. *J Chromatogr B Analyt Technol Biomed Life Sci* **2003**, *785*, 263–275.
